# Nanotechnology in Retinal Disease: Current Concepts and Future Directions

**DOI:** 10.1089/jop.2023.0083

**Published:** 2024-02-09

**Authors:** Nalin J. Mehta, Sachin N. Mehta

**Affiliations:** ^1^ Colorado Retina Center, Lakewood, Colorado, USA.; ^2^ Johns Hopkins University, Baltimore, Maryland, USA.

**Keywords:** biotechnology, retina, drug delivery, nanotechnology, gene therapy, ophthalmology

## Abstract

The retina is one of the most complex and extraordinary human organs affected by genetic, metabolic, and degenerative diseases, resulting in blindness for ∼1.3 million people in the United States and over 40 million people worldwide. This translates into a huge loss of productivity, especially among younger patients with inherited retinal diseases (IRDs) and diabetic retinopathy. Age-related macular degeneration accounts for 90% of all blindness cases worldwide. The prevalence of this condition is projected to reach over 5 million individuals over the next 3 decades. There are also >20 IRD phenotypes, affecting >2 million people worldwide. Nanobiotechnology uses nanotechnology for biological applications, making use of biological materials either conceptually or directly in the fabrication of new materials. Bionanotechnology, on the other hand, uses molecular biology for the purpose of creating nanostructures (ie, structures with at least 1 dimension <100 nm). Retinal applications of these technologies are developing at a rapid pace. This review includes the most current nanotechnological applications in retinal diagnostics, theranostics, drug delivery, and targeting, including the potential for nonviral vehicles such as liposomes, micelles, and dendrimers, which pose advantages over viral vectors in retinal drug delivery. Furthermore, we discuss current and future applications as surgical adjuncts and in regenerative medicine as they pertain to retinal disease. Structure and function of nanoparticles such as carbon nanotubules, quantum dots, and magnetic nanoparticles, as well as diagnostic technologies such as next-generation DNA sequencing and single-molecule bionanosensing, will also be discussed.

## Background

**FIG. 1.**
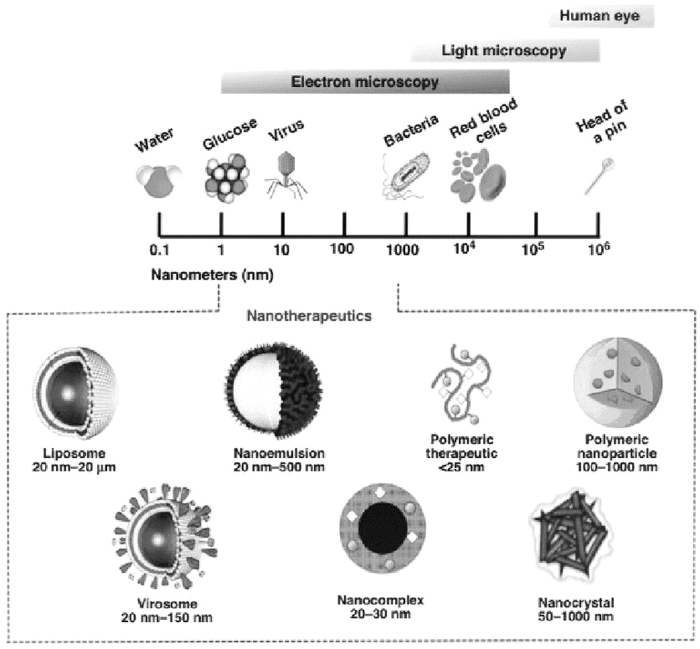
Nanoscale comparison and types of nanotherapeutics used in drug delivery. Reprinted with permission from Maria Malamatari, MPhar, PhD, AFHEA.^1^

Nanotechnology is defined as technology on the scale of 1 to 100 nm in at least 1 dimension (Fig. 1).^1^ At this scale, the laws of gravity and inertia are replaced by charge–charge interactions and quantum mechanics; fluid flow loses its turbulence; photonic properties become size dependent; and particles become highly reactive because of their enormous surface-to-volume ratios.

Nanobiotechnology can prove useful in retinal diagnostics and pharmacological and surgical therapeutics, as well as drug delivery (Tables 1 and 2). Retinal regenerative medicine also stands to benefit from nanoscience. The diminutive size of the retina, where therapeutics and surgery are in the range of micrograms and microns, respectively, combined with its relative immune privilege, and easy accessibility for both observation and treatment make it ideally suited for nanobiotechnological innovations.

**Table 1.** Potential Retinal Nanodiagnostics

Diagnostics	Nanoparticle	Model	Results
NP-enhanced optical coherence tomography	Au nanoparticles^2^ Fe-TiO_2_ nanoparticles^3^	Rabbit skin^2^ Chicken breast^3^	Skin layer border contrast^2^ 210–780-μm penetration^3^
NP-enhanced magnetic resonance imaging	Gd-PFCL emulsion linked with biotinylated antibody^7^	Rabbit corneal neovascularization	Enhances magnetic resonance signal intensity in the corneal capillary bed
Atomic force microscopy	Rhodopsin molecule visualization^8^	Murine rod outer-segment disc membranes *in vitro*	Rhodopsin molecule stability is a function of strength of interconnected segments
Photoacoustic microscopy	Oxygen metabolic studies^9^ Choroidal imaging^10^	Rat^9^ Rat and rabbit^10^	O_2_ saturation measurements^9^ Measurement of melanin, O_2_ saturation, and blood flow^10^
Bionanosensors	Gold nano-interdigitated arrays^12^	*Ixodes ricinus* im munosuppressor (anti-iris) protein	Detection of protein–antibody reaction at a concentration <1 ng/mL
Biologic assays	Q-dots and antibody-functionalized MNPs^11^	Nanotechnology-on-a-chip	Multicolor optical coding; potential for identification of pathologic substances *ex vivo* and *in vitro*
Nanopore gene sequencing	cDNA-functionalized AuNPs^11,13^	Nanotechnology-on-a-chip	Potential identification of inherited retinal diseases

AuNPs, gold nanoparticles; GD-PFCL, gadolinium–perfluorocarbon liquid; MNPs, magnetic nanoparticles; NP, nanoparticle; Q-dots, quantum dots.

**Table 2.** Current and Potential Retinal Nanotherapeutics and Theranostics

Retinal application	Nanoparticle	Model	Conclusion
Precision cell ablation (ie, tumor cells)	AuNPs^14^	Various cellular targets	Aggregate AuNPs enhance detection and treatment over single nanoparticles
Gene therapy	AuNPs^15^	Various plasmid, minivector DNA, and siRNA vectors	AuNPs enhance nucleic acid delivery
AMD	Antiangiogenic peptides^19^	Murine	Angiogenesis decreased for at least 14 weeks after a single dose
AMD	Cell-penetrating peptides^20^	Rat	Single dose improves retinal permeability, increases antioxidant retention, and suppresses neovascularization for 56 days
Ocular tumors	Functionalized Q-dots^16^	Human osteosarcoma cells	For single-cell microscopy, cells exhibit strong fluorescence and hypersensitivity; Q-dots are nontoxic and biologically inactive
Ocular tumors	MNP hyperthermia^17,18^	Zebrafish embryos^17^ Humans^18^	Functionalized MNPs preferentially localized to the choroid and retinal pigment epithelium ^17^ Thermotherapy using MNPs was proven safe and effective^18^
AMD	Topical nanoemulsions^22–24^	Primate^22^ Human embryonic kidney cells and rat Müller cells^23^ Rats^24^	Drug in eyedrop form was suggested to penetrate both the cornea and blood–retinal barrier to reach the fundus^22^ Improved long-acting intraocular bioavailability of hydrophobic celecoxib^23^ Enhanced retinal accumulation of anti-VEGF apatinib^24^
AMD	Dendrimers^28^	Human donor eyes and rats	Pathology-dependent biodistribution; suppression of choroidal neovascularization; and cytokine suppression
AMD	Self-assembling polymeric micelles^25^	Various *in vitro* and *in vivo* models	Improved bioavailability, bioactivity, intracellular penetration, controlled delivery, and retention time
AMD and inherited retinal diseases	Viral	Human subjects	RGX-314, ADVM-022, and voretigene neparvovec-rzyl
AMD	Self-assembling polymers^29^	Human subjects	Targeted delivery of siRNA
AMD	Nanoceria^62^	Human ARPE-19 and umbilical endothelium cell lines	Anti-inflammatory, antiangiogenic, and antiapoptotic properties

AMD, age-related macular degeneration; siRNA, small interfering RNA; VEGF, vascular endothelial growth factor.

## Diagnostics

Current modalities of retinal imaging, including optical coherence tomography (OCT), ultrasonography, and angiography, can all benefit from nanotechnological innovation, with the potential to combine diagnostic modalities with therapeutic intervention (ie, theranostics).

Commercial OCT technologies have axial resolutions approaching <4 microns, with scan speeds approaching 80,000 scans/s, which allow for retinal angiographic imaging without the assistance of intravenous dyes. One of the challenges with this technology, however, is that although fine neovascular structures can be identified, the specificity can be limited by optical artifacts from adjacent vascular structures. Retinal angiography, moreover, can demonstrate chorioretinal vascular leakage, whereas OCT angiography, lacking contrast agents, is unable to demonstrate leakage directly.

One solution being investigated is the use of gold nanoparticles (AuNPs) as contrast agents for OCT, given the ability to create particles small enough to pass through even the smallest retinal or choroidal vessels (almost 3 microns in diameter) with wavelength absorption efficiencies higher than conventional angiographic dyes.^2^ Iron–titanium dioxide nanoparticles have also been investigated as contrast agents in swept-source OCT.^3^

The use of ultrasonography in the diagnosis of retinal pathology has been widespread for decades. Using frequencies of 6–20 MHz, ocular ultrasonography has a resolution that is an order of magnitude lower than OCT.^4^ The use of microbubbles of gas as an ultrasonic graphic contrast agent was first reported by Gramiak and Shah in the 1960s as an adjunct to echocardiography.^5^

One concern with this technique for ophthalmic use on a nanoscale is creating nanosized bubbles with size consistency and stability, as too large a gas bubble in a small capillary could result in sight-threatening vascular occlusion. In addition, longevity is an important factor as they would need to be sustained from the time of administration to imaging.

Perhaps long-acting gases such as perfluoropropane (C_3_F_8_) or sulfur hexafluoride (SF_6_), currently used for long-term postoperative retinal tamponade during retinal detachment surgery, may be able to fulfill this requirement.

MRI on its own has low sensitivity for microscopic retinal structures. To enhance corneal neovascular diagnostic sensitivity, Anderson et al. have developed a gadolinium–perfluorocarbon nanoparticle emulsion linked with a biotinylated antibody. Given that perfluorocarbon liquids are radiopaque, this combination would allow for targeted enhancement of neovascularization using MRI.^6,7^ Similar technology could be applied to the retinal and choroidal vasculature for enhanced imaging of neovascularization associated with age-related macular degeneration (AMD) or vascular neoplasms, such as choroidal melanomas.

Visualization of biomarkers on a molecular level requires nanoscale imaging techniques. Atomic force microscopy (AFM), with subnanometer resolution, is able to fulfill this role. AFM detects changes in the reflective angle of a laser beam focused upon a nanoscale cantilever as it traces the surface of subcellular structures, creating three-dimensional surface profiles not only of retinal cell and organelle membranes but also nanostructures such as rhodopsin molecules.^8^

Photoacoustic microscopy (PAM) allows for not only structural but also functional assessment of the retina and choroid on a molecular level by using high-resolution identification of endogenous chromophores such as hemoglobin and melanin. PAM detects the thermoelastic expansion of chromophores derived from rapid temperature changes generated by single-wavelength nanopulse laser absorption; an ultrasound transducer is used to detect the resulting photoacoustic waves. PAM can be integrated with OCT for enhanced chorioretinal localization of oxygen saturation measurements.^9,10^

Another facet of diagnostics involves identification of the hundreds of genetic variants that may contribute to inherited retinal diseases (IRDs). Treatments are actively being investigated for several of these IRDs such as Stargardt disease and retinitis pigmentosa. However, current point-of-service (POS) testing relies on saliva samples that are then sent to an off-site laboratory for analysis utilizing amplification techniques such as polymerase chain reaction and subsequent mass spectroscopy, which take days to perform. Next-generation sequencing (NGS) holds promise for more sensitive, efficient, and portable diagnostics, which can be utilized in an office setting with rapid results.

Nanoscale molecular diagnostics can revolutionize detection capabilities and sensitivities. Magnetic nanoparticles (MNPs) functionalized with antibodies, for example, can be used for localization of specific intracellular molecules, which could aid in identification of pathologic accumulation of materials [ie, lipofuscin in retinal pigment epithelium (RPE) cells].

When attached to segments of complementary DNA, AuNPs can identify genetic abnormalities in cell samples obtained from relatively noninvasive extraocular sources as well as multicolor coding in biologic assays using quantum dots (Q-dots)^11^—semiconducting molecules with diameters from 2 to 10 nm and the ability to fluoresce in pure long-lasting colors when excited with white or UV light, which in turn transfers electrons from low-energy valence bands to high-energy conductance bands. These electrons then drop back into their respective valence bands, releasing photonic energy at specific wavelengths while doing so.

Portability could be achieved with lab-on-a-chip-type technologies incorporating bionanosensors utilizing various nanoscale technologies. For instance, functionalized carbon nanotubules (CNTs) with diameters as small as 1 nm, as well as gold nano-interdigitated arrays with digit widths as narrow as 10 nm, can be incorporated into transistor-like circuitry for single-molecule detection by functionalizing the surface with antibodies to bind to target DNA or other biologic molecules, thereby changing surface electrical conductivity.^12^

Similarly, nanopore DNA sequencing takes advantage of changes in conductivity when DNA nucleotides are sequentially passed through nanopores that are ∼2 nm in diameter and embedded in a protein sheet. Combined with nanofluidics, nanopore sequencing can be used to perform rapid, real-time DNA sequencing.^13^ The goal of these NGS technologies would be to eliminate the need for time-consuming transport and amplification of biologic samples by increasing diagnostic sensitivity at the molecular level and decentralizing the analysis to POS locations.

## Theranostics

Functionalization of AuNPs can increase specificity not only for diagnostics but also theranostics. AuNPs can easily be heated using radiofrequency waves, destroying cells into which the AuNPs are introduced with a precision critical for small treatment spaces with minimal tolerance for collateral cell damage.

AuNPs can also be used for delivery of DNA and RNA for gene therapies, given their ability to bind with synthetic and biological compounds.^14,15^ Using this nonviral vector for gene delivery could alleviate some of the challenges associated with current adeno-associated viral (AAV) vectors—notably inflammation and payload size limitation—maximizing therapeutic effects by allowing for increased potency.

The fluorescence characteristics of Q-dots can be customized by varying particle size and, when functionalized with therapeutic agents, they can target ocular tumors for both diagnostic imaging and treatment.^16^

Functionalized MNPs may also be useful for identifying ocular tumors using techniques such as OCT or CT scanning; targeted drug delivery to the choroid and RPE; and precision MNP hyperthermia using alternating magnetic fields to induce high-frequency oscillations of MNPs, causing frictional heating and subsequent tumor destruction.^17,18^

CNTs or fullerenes (ie, buckyballs, naturally occurring carbon-60 spheres) functionalized with both therapeutic agents [ie, AuNPs and nanoceria (a “nanoenzyme” derived from cerium oxide, which has proven to be both antiangiogenic and anti-inflammatory)] and biomarkers (ie, fluorescent nanosensors) could create powerful theranostic tools for simultaneous diagnosis and treatment of retinal pathologies.

## Therapeutics: Drugs, Delivery Systems, and Targeting

Nanobiotechnology has already made great strides with regard to drug delivery in the eye. Given the minute dimensions involved, the ideal therapeutic will have small volume, high potency, and accurate localization—these are the advantages to be gained by nanoscale formulations.

One of the challenges with retinal drug delivery involves the ability of the drug to migrate from the preretinal to retinal or even subretinal spaces. Analogous to the blood–brain barrier, the blood–retina barrier (BRB) prevents blood from entering extravascular spaces. Unfortunately for drug delivery, these barriers are efficient in keeping out therapeutic substances deemed foreign. However, with the help of nanotechnology, these barriers can be successfully circumvented.

Intravitreal drug delivery through injection is currently a mainstay of retinal treatments for conditions such as diabetic retinopathy and exudative AMD. Vascular endothelial growth factor (VEGF) inhibitors are antibody sized (∼10 nm); these medications readily penetrate the retina to enter the subretinal space, where choroidal neovascularization originates in patients with exudative AMD.

Intravitreally delivered agents other than anti-VEGFs also show promise, such as antiangiogenic peptides; to prevent degradation before delivery, self-assembled nanoparticles are subsequently coated to form poly(lactic-co-glycolic acid) (PLGA) nanoparticles with slow-release properties.^19^

**FIG. 2.**
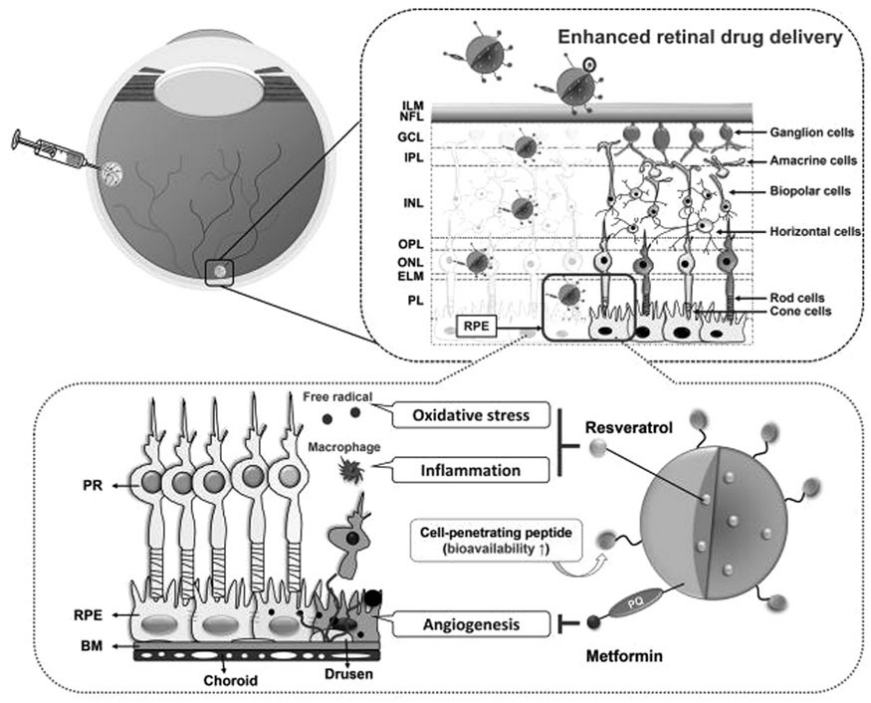
Schematic representation of the pharmacological treatment of the neovascular AMD eye using PCL NPs as nanocarriers; a cell-penetrating peptide as a retinal penetration enhancer; and resveratrol and metformin as model drugs for synchronic attenuation of oxidative stress, inflammation, and angiogenesis in the diseased retina. Reprinted with permission from Nguyen et al.^20^ AMD, age-related macular degeneration; BM, Bruch's membrane; ELM, external limiting membrane; GCL, ganglion cell layer; ILM, inner limiting membrane; INL, inner nuclear layer; IPL, inner plexiform layer; NFL, nerve fiber layer; NPs, nanoparticles; ONL, outer nuclear layer; OPL, outer plexiform layer; PCL, poly(E-caprolactone); PL, photosensitive layer; PR, photoreceptors; RPE, retinal pigment epithelium.

Another synthesized nanoparticle utilizes the biodegradable biopolymer, poly(E-caprolactone) (PCL), embedded with resveratrol, which demonstrates antioxidant and anti-inflammatory properties. The surface is coated with a combination of metformin, which is known to inhibit choroidal neovascularization, and cell-penetrating peptides, which increase retinal permeability by 15-fold after intravitreal injection (Fig. 2).^20^

Intravitreal administration of gene therapies is also being investigated using AAV vectors (∼20 nm in size) containing DNA, which codes for VEGF-inhibiting medication production (ie, REGENXBIO RGX-314; Adverum Biotechnologies ADVM-022). Given the aforementioned limitations of viral vectors, most notably inflammation, nonviral gene delivery would seem to be a favorable alternative.

Transcorneal and transconjunctival nanoemulsions are much less invasive and can enhance delivery and retention of poorly soluble drugs through these layers and into the eye through endocytosis, delivering high potency because of the high nanoparticle surface area-to-volume ratio.^21^ A nanoparticle formulation of aminocaproic acid-modified Dio is able to penetrate both the cornea and BRB, resulting in antiangiogenic activity with low cellular toxicity in a primate AMD model.^22^

Similarly, topical celecoxib-loaded poly(ortho ester) nanoparticles have been formulated, possessing both anti-inflammatory and antiproliferative properties.^23^ Researchers have also engineered serum albumin nanoparticles containing apatinib (an anti-VEGF receptor 2 tyrosine kinase inhibitor), which when coated with hyaluronic acid, are able to penetrate the cornea and target retinal cells.^24^

Other mechanisms of ocular delivery of retinal drugs could include polymeric nanoparticles because of their favorable biocompatibility and biodegradability as well as self-assembling polymeric micelles of small size (as small as 5 nm), water solubility, and controlled drug release.^25,26^

Dendrimers are highly branched molecules (as small as 1.5 nm in diameter) that can allow for codelivery of multiple drugs as well as effect sustained delivery. Dendrimer technology is already being used for glaucoma drug delivery.^27^

Liposomes (the water-insoluble “opposite” of micelles) and lipid nanoparticles (LNPs) are additional options. Although most of these strategies are being used for corneal/anterior segment disease, they could be utilized to provide noninvasive drug delivery to the vitreoretinal/subretinal structures.

Systemic delivery of retinal therapeutics is also possible. The challenge, however, is delivering the drug to target tissues in the eye while avoiding systemic degradation during transit; in addition to alteration of pharmaceutical properties, a proteinaceous biocorona can form around the active molecule, potentially altering both its pharmacologic and pharmacokinetic properties.

Systemic dendrimer-based molecules have demonstrated selective, passive RPE uptake of steroid in damaged cells, which can suppress both inflammation and choroidal neovascularization.^28^ Next-generation gene therapies, such as small interfering RNA, can be delivered through self-assembled polymers to which both targeting and therapeutic agents can be attached; such polymers may be suitable for systemic administration, having low toxicity and immunogenicity.^29^

MNPs have been used to accurately deliver induced pluripotent stem cells (iPSCs) to the trabecular meshwork *in vivo*; similar methods could potentially be used for iPSC-derived RPE delivery.^30^

## Surgical Adjuncts

Despite improvements in microscope optics and resolution, and even with a surgeon's steady hand, a limiting factor in retinal surgery appears to be instrument size. Investigators are attempting to design nanoscale instruments for nanosurgical repairs. Nanotube tweezers have been designed by fusing 2 carbon nanotubes with a spacing of 10 nm.^31^ Others have proposed direct nerve fiber axon repairs using a knife edge with a 20-nm radius of curvature, with attachment of a transplanted axon segment performed by electrofusion.^32^ Current instruments can be coated with silver nanoparticles that confer both anti-infective and antioxidative properties.^33^

YAG laser vitreolysis, a relatively new technique that can serve as an alternative to pars plana vitrectomy for visually debilitating vitreous floaters, has been facilitated by the development of newer laser delivery systems; however, the relatively high-power settings pose potential complications with vitreous opacities close to the retina or lens. Hyaluronic acid-coated AuNPs have been found to cluster on vitreous opacities *in vivo*.

Low-energy, nanosecond laser pulses can subsequently be applied to create vitreous nanobubbles by ultrafast heating of AuNPs that in turn ablate these vitreous opacities with significantly reduced energy levels when compared with conventional YAG vitreolysis.^34^

## Nanoprostheses

Retinal implants continue to evolve, from the Argus II (Second Sight Medical Products) and IRIS II (Pixium Vision), with epiretinal microelectrode arrays of up to 60 electrodes and external hardware, to the compact and self-contained Alpha MS (Retina Implant AG), which utilizes a subretinal photodiode array of 1,500 electrodes to detect light and deliver a charge to the inner retina.^35,36^ Even as implants become more compact, the best visual resolution is still in the 20/400 range, a limitation of sensor resolution; the technology is also quite expensive.

With the smallest nanoscale transistors now only 1 nm in length and diodes as small as 1 molecule in size, we would expect resolution to increase in the future, with a corresponding decrease in the intrusiveness and cost of the implant. Another promising technology involves poly(3-hexylthiophene) (P3HT) nanoparticles injected into the eye. These nanoparticles are injected in liquid form into the subretinal space, mimicking the spatial distribution of photoreceptors and forming a light-sensitive interface.^37^

CNT nanoprosthetics are being integrated with neural tissue to guide synaptic development during neuronal repair.^38^ Such technology could theoretically help patients with neuro-ophthalmologic trauma or degeneration.

## Regenerative Nanobiotechnology

Diseases such as macular degeneration and diabetic retinopathy result in premature aging, trauma, and inflammation of retinal tissues, with cell loss from oxidative stress and subsequent apoptosis. Future therapies should therefore include the ability to not only protect retinal tissues from these ravages but also to repair and regenerate structure and function. Metallic and metal oxide nanoparticles are uniquely equipped to help with inflammation and oxidative stress.

AuNPs are not only relatively inert but also possess intrinsic anti-inflammatory and antiangiogenic properties.^39^ Nanoceria is another powerful example of a reactive oxygen species scavenger. In addition, nanoceria can help to both promote and regulate healthy angiogenesis vital for regenerating tissues while demonstrating anti-VEGF properties consistent with its role in angiogenic modulation without adverse effects on vision.^40^

Dexamethasone-conjugated dendrimers (D-Dex) demonstrate selective affinity for damaged retinal Müller glial cells, promoting regenerative stem cell-like properties in mammals. The selectivity of D-Dex, furthermore, minimizes the potential for systemic toxicity.^41^

CNTs, described previously, are not only useful in diagnostics but can also inherently function as free radical scavengers, potentially reducing oxidative stress thought to play a vital role in progression to wet AMD. Similarly, fullerenes are inherently antioxidative and anti-inflammatory.^42^

In addition to previously mentioned theranostics, MNPs are being used for targeted stem cell delivery within the eye. Yanai et al. describe a technique of magnetizing rat mesenchymal stem cells using superparamagnetic iron oxide nanoparticles and, after intravenous injection, inducing migration and localization to the inner and outer retina with a magnet placed in the orbit.^43^

Developing viable scaffolding is essential to retinal regeneration as both primary stem cells and iPSCs rely upon these scaffolds for proper orientation. With proper growth and differentiation/dedifferentiation factors, as well as niche formation allowing for the proper juxtaposition of cellular and extracellular matrix components, retinal regeneration has a higher probability of success. Nanoscaffolds are being developed to aide in retinal tissue regeneration.

Repopulation with normal retinal cells becomes challenging because of the multilayered nature of the retina as well as the intricate interconnectivity between different cells. Natural nanofiber scaffolds are being created from materials such as gelatin, chitosan, collagen, and hyaluronic acid. These have been shown to promote healthy RPE growth and release of regenerative factors, with superior cellular adhesion to that of synthetic scaffolds created from biodegradable polymers such as PLGA, although the latter have better strength and therefore longer half-lives.^44^

Electrospinning is a technique whereby nanofibers are created by extrusion of a polymer solution that is then carried to a distant electrically charged plate (analogous to a cotton candy machine, except using electrostatic rather than centrifugal forces). This technique has recently been used to create a nanofiber scaffold upon which RPE cells can be cultured with the possibility of subsequent subretinal transplantation.^45^

## Future Directions

The future is exceedingly bright for nanobiotechnological applications in ophthalmology. With technologies such as clustered regularly interspaced short palindromic repeats (CRISPR) combined with the enzyme, CRISPR-associated protein 9 (CRISPR-Cas9), gene editing has the ability to address multiple genes simultaneously, which could prove beneficial for multifactorial diseases such as AMD, for which over a dozen single-nucleotide polymorphisms have been found to be associated with risk of disease progression.

AAV vectors are currently the most common means for retinal intracellular delivery of genetic material; however, associated risks include immunogenic responses, as mentioned previously, and off-target genomic editing contributed by the sustained presence of Cas9; pre-existing immunity to this persistent Cas9 can also negate CRISPR gene editing.^46,47^

These issues can potentially be avoided by using nonviral delivery methods; however, transfection efficiency is limited by the lack of effective delivery systems, and successful delivery of CRISPR-Cas9 to the outer retinal cells *in vivo* continues to elude researchers.^48^ Current methods that are under investigation and could potentially be utilized for CRISPR-Cas9 delivery to the retina *in vivo* include nonchemical modalities such as sonoporation, where cell membrane permeability is increased by creating pores using ultrasound irradiation to create microbubbles.

However mechanical and physical disruption of the cell membranes, however transient, risks long-term trauma to the cells with subsequent adverse effects on vision. There are also challenges in isolating target retinal cells given the microscopic dimensions as well as intricate juxtaposition of various retinal cellular components.^49,50^

Chemical modalities include AuNPs, LNPs, and polymeric nanoparticles, whose positive charge readily interacts with negatively charged DNA.^48,51^ Carefully designed inorganic nanoparticles can overcome the challenges of crossing the BRB, inefficacy from rapid degradation, and both gene and cellular toxicity from overpersistence. Such nanoparticles include polymers, silicon, and organometallic composites.^51^

iPSC-derived RPE cells can be derived from fibroblasts or mononuclear blood cells by incubation with various protein-coding genes such as *LEFTY2*.^52^ Müller glial cells, furthermore, have been shown to transdifferentiate to rod photoreceptors using the sonic hedgehog gene.^53^ Targeted delivery of messenger RNA (mRNA) to the RPE, Müller glia, and neural retina has been accomplished using LNPs, which can overcome limitations of AAV vectors.^46,54^

Delivery has, however, been limited to direct subretinal injection, which is highly invasive, or intravitreal injections that are nonlocalizing. Laser-enhanced delivery of genetic material is a novel technique utilizing either direct or indirect optoporation in precise areas of retinal degeneration after intravitreal injection.^55,56^ Similar methodology could conceivably be used to deliver gene-laden LNPs or other nonviral nanoparticles.

Exosomes are bilayered nanovesicles that range upward from 30 nm in size, originating from invagination of plasma membranes. They show promise as disease biomarkers, intercellular communication vehicles, and drug delivery vehicles.^57^ MicroRNA (miRNA) plays an important role in both cellular maintenance and inflammatory modulation. Exosomes have been shown to transport miRNA between RPE cells and retinal glial cells.

This finding could prove useful in modulating senescence and apoptosis of the neuroretina in conditions such as AMD, where RPE cells show an increase in exosomal production with oxidative stress, and diabetic retinopathy, where a similar increase in exosomal production occurs in retinal glial cells.^58^

**FIG. 3.**
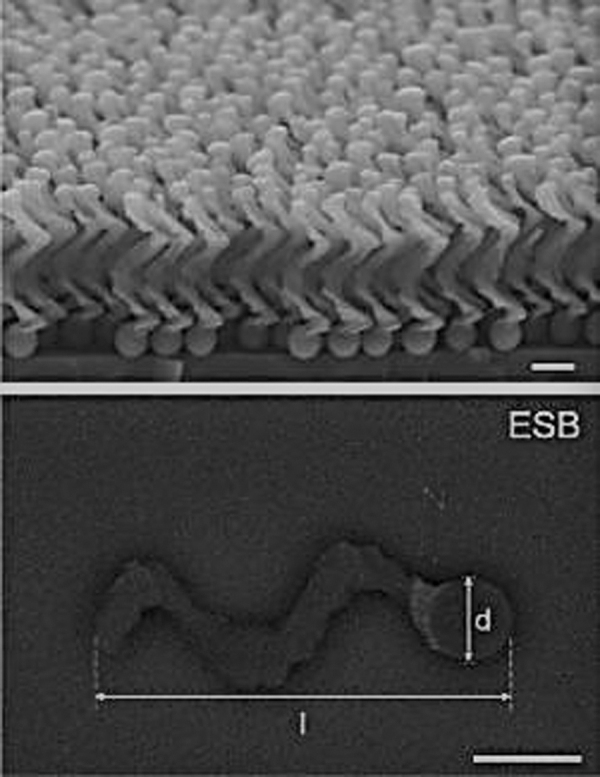
SEM (*top*) and ESB-SEM (*bottom*) images of micropropellers. Scale bar = 500 nm. Reprinted with permission from Wu et al.^59^ ESB-SEM, energy selective backscatter-scanning electron microscope.

Researchers have created helicoid, magnetic nanoscale micropropellers that are guided by external magnetic fields to traverse the vitreous cavity, with the potential to deliver therapeutics to the retina with precision (Fig. 3).^59^ With a diameter of 500 nm, these nanorobots are relatively large; however, given the ability to fabricate structures such as functionalized DNA nanostructures with MNPs, rather than with dyes and other nanomaterials currently being used for ultrasensitive molecular imaging, it is conceivable that such nanorobots could be engineered to obtain diameters approaching 2 nm.^60^

Recent innovations in passive, nonviral, targeted retinal drug delivery (thereby minimizing both invasiveness and inflammation) include the development of a polyacrylamide nanoparticle (ANP) platform integrated with neuroprotective neurotrophin nerve growth factor and utilization of the targeting agent, peanut agglutinin, which has an affinity for retinal rods and cones.

This polymer-based nanoparticle has been shown to prevent retinal cell apoptosis characteristic of retinal degenerative diseases such as AMD.^61^ Similarly, oligochitosan-coated nanoceria demonstrates antiangiogenic, anti-inflammatory, and antiapoptotic characteristics in AMD cellular models.^62^

LNPs functionalized with targeting peptides conjugated with mRNA are now able to bypass retinal barriers that have, to date, limited LNP penetration into RPE and Müller glial cells, allowing the functionalized LNPs to reach photoreceptors, a critical target in designing gene therapies for IRDs.^46^

Reengineering the photoreceptors themselves has also been a topic of recent research. Sp et al. have succeeded in nanoparticle-mediated delivery of a full-length, human rhodopsin gene (*gRHO*) to murine rod photoreceptors, resulting in decreased degeneration; this could be useful in future treatment of retinitis pigmentosa.^63^ Kwon et al. have synthesized melanin-like nanoparticles with powerful antioxidant properties, which could be utilized as an artificial melanin substitute in murine RPE cells, in turn stemming deterioration seen in AMD.^64^

We have only begun to scratch the surface of what is possible with therapeutic and regenerative applications of nanotechnology in the field of retina.

Finally, the ability to engineer nanostructures at an atomic level using devices such as the scanning tunneling microscope (which utilizes the tunneling phenomenon of quantum mechanics to not only image subnanometer structures but also move individual atoms^65^) will open the door to an entirely new class of nanotherapeutics, where nanoscale circuits as well as pumps, such as those existing across cell membranes where they are utilized for transmembrane transport, could be fabricated and utilized in diagnostic, maintenance, and restorative functions; indeed, the best is yet to come.
